# Microglial Priming in Infections and Its Risk to Neurodegenerative Diseases

**DOI:** 10.3389/fncel.2022.878987

**Published:** 2022-06-15

**Authors:** Maiara N. Lima, Maria C. Barbosa-Silva, Tatiana Maron-Gutierrez

**Affiliations:** ^1^Laboratory of Immunopharmacology, Oswaldo Cruz Institute, Oswaldo Cruz Foundation, Fiocruz, Rio de Janeiro, Brazil; ^2^National Institute of Science and Technology on Neuroimmunomodulation, Rio de Janeiro, Brazil

**Keywords:** pro-inflammatory microglia, microglial priming, infectious diseases, neurodegenerative diseases, aging, central nervous system inflammation, brain inflammation

## Abstract

Infectious diseases of different etiologies have been associated with acute and long-term neurological consequences. The primary cause of these consequences appears to be an inflammatory process characterized primarily by a pro-inflammatory microglial state. Microglial cells, the local effectors’ cells of innate immunity, once faced by a stimulus, alter their morphology, and become a primary source of inflammatory cytokines that increase the inflammatory process of the brain. This inflammatory scenario exerts a critical role in the pathogenesis of neurodegenerative diseases. In recent years, several studies have shown the involvement of the microglial inflammatory response caused by infections in the development of neurodegenerative diseases. This has been associated with a transitory microglial state subsequent to an inflammatory response, known as microglial priming, in which these cells are more responsive to stimuli. Thus, systemic inflammation and infections induce a transitory state in microglia that may lead to changes in their state and function, making priming them for subsequent immune challenges. However, considering that microglia are long-lived cells and are repeatedly exposed to infections during a lifetime, microglial priming may not be beneficial. In this review, we discuss the relationship between infections and neurodegenerative diseases and how this may rely on microglial priming.

## Introduction

Infections of different etiologies, neurotropic or not, have been associated with acute and long-term neurological consequences (Jurgens et al., 2012; Hosseini et al., 2018; Barbosa-Silva et al., 2021). These consequences involve cognitive decline and behavioral disorders such as depression and anxiety. The main cause of these sequelae is an inflammatory condition in the central nervous system (CNS) characterized by an increase in pro-inflammatory mediators secreted by glial cells, such as microglia and astrocytes (Dantzer et al., 2008; Wendeln et al., 2018).

Microglia, which has long been described as a resident immune cell in the CNS, is currently considered an essential and versatile cell, having well-defined roles in maintaining neuronal networks, supporting synaptic plasticity, repairing injuries, and participating in the inflammatory process (Heneka et al., 2015). Microglial cells express pattern recognition receptors (PRRs) that recognize molecules known as pathogen-associated molecular pattern molecules (PAMP) and damage-associated molecular patterns (DAMPs). During infection, irrespective of whether the pathogen can invade the CNS, the microglia will respond quickly by altering its state. Once confronted with stimuli, microglia induce and modulate a broad spectrum of molecular and cellular responses in an attempt to eradicate the pathogen (Heneka et al., 2014; Widmann and Heneka, 2014).

Evidence suggests that changes in microglial morphology, functionality, and subsequently priming of microglial cells may be involved in the development of neurodegenerative diseases (Perry et al., 2007; Püntener et al., 2012; Barbosa-Silva et al., 2018; Haley et al., 2019; De Sousa et al., 2021). In this review, we will focus on microglial changes resulting from infectious diseases inducing microglial priming and how this phenomenon could be associated with the development of neurogenerative diseases.

## Microglia: from Homeostatic to Pro-Inflammatory State

Microglia are currently defined as yolk sac-derived, long-living cells that persist into adulthood and self-renew within the CNS parenchyma without any contribution from bone marrow-derived cells in the steady-state (Paolicelli et al., 2022). Microglial cells are heterogeneous and vary according to the brain region, displaying distinct intrinsic properties and performing distinct physiological functions. They can vary with age, sex, and pathology-specific (or stimulus-specific) (Davalos et al., 2005; Hines et al., 2009; Lenz and McCarthy, 2015; De et al., 2018; Plescher et al., 2018; Stratoulias et al., 2019; Villa et al., 2019). Due to their myeloid origin, microglia have long been described as CNS macrophages. However, other populations of CNS macrophages can be found, such as perivascular macrophages, meningeal macrophages, circumventricular organ macrophages, and choroid plexus macrophages (Kierdorf et al., 2019). Although microglia and macrophages share common characteristics, such as a strong and variable capacity to respond to inflammatory conditions, they differ in ontogeny, number, function, and mainly in location (Kierdorf et al., 2019).

Microglia are anything but static, as they are extremely sensitive to changes in their environment (Paolicelli et al., 2022). A broader spectrum of different microglia states is associated with different stimuli and roles in homeostasis and disease (Streit, 2002; Herz et al., 2017; Tay et al., 2017; Muzio et al., 2021). Under homeostatic conditions, microglia present a homeostatic state that participates in several active functions within the CNS, including continuous motility and maintenance of CNS functions. Their functions evolve in response to their specific location and reciprocal interactions with nearby cells and structures and play essential roles, such as neurotrophic factor production, synaptic pruning, and immunological surveillance (Nimmerjahn et al., 2005; Stratoulias et al., 2019; Paolicelli et al., 2022). Their morphology, ultrastructure, and molecular profile are all dynamic and plastic, resulting in a wide range of cell states (Paolicelli et al., 2022).

Hippocampus neurogenic niches and the production of new neurons also depend on roles played by homeostatic microglial cells (Arnò et al., 2014). Additionally, these cells are necessary for the elimination of amyloid β (Aβ) peptide and abnormal tau protein, protecting the CNS from the development of neurodegenerative diseases (Bellucci et al., 2004; Hickman et al., 2008; Meyer-Luehmann et al., 2008; Sasaki et al., 2008; Zilka et al., 2009; Nalivaeva et al., 2012; Ries and Sastre, 2016; Perea et al., 2018a, 2020; Španić et al., 2019; Jin et al., 2021; d’Errico et al., 2022). The morphology of the homeostatic microglia presents cellular processes with high motility, which help to detect the brain parenchyma, and interact with other cells, such as neurons and astrocytes, and blood vessels (Nimmerjahn et al., 2005; Hickman et al., 2018; Stratoulias et al., 2019). Furthermore, these states are characterized by constitutive expression of macrophage antigens such as complement receptor 3 (CD18/CD11b) and low expression of the major histocompatibility complex (MHC) II (Ehlers, 2000; Frank et al., 2006; Colton and Wilcock, 2010; Czirr et al., 2017).

Conversely, during CNS injuries, such as infectious and neurodegenerative diseases, microglial cells present a complex response, transitioning from a homeostatic state and assuming a pro-inflammatory state to solve the infection/injury. Among the states that can be assumed by microglia is an anti-inflammatory state, whose function is to promote tissue remodeling and repair although, in several cases, microglia assume a pro-inflammatory state. It is not easy to accurately define these states because the responses are highly heterogeneous and microenvironment dependent. Even similar stimuli may elicit distinct microglia responses, constructing a different spectrum of reactivities (Scheffel et al., 2012; Kamigaki et al., 2016; Friedman et al., 2018; Furube et al., 2018; Stratoulias et al., 2019). Despite differences, it is possible to define characteristics and roles associated with a pro-inflammatory microglial state that characterizes inflammatory conditions. The acute inflammatory response leads to functional and morphological changes in microglial cells, including upregulation of some molecules, such as CD11b, Intercellular adhesion molecule-1 (ICAM-1), P-selectin, major histocompatibility complex II (MHC II), CD80 (T-cell costimulatory molecules B7–1), CD86 (T-cell costimulatory molecules B7–2), and CD40 [a member of the tumor necrosis factor (TNF) receptor; Colton and Wilcock, 2010; Yeini et al., 2021]. Pro-inflammatory microglia cells present a branch architecture with thicker processes and in some cases a complete amoeboid morphology, characterized by a round cell body and few and short processes, similar to a macrophage (Davis et al., 1994; Fernández-Arjona et al., 2017; Savage et al., 2019; Franco-Bocanegra et al., 2021). Furthermore, they increase the production and release of mediators, including reactive oxygen species (ROS), interleukin (IL)-1β, IL-6, TNFα, nitric oxide (NO_2_), acute phase proteins such as pentraxin-3 involved in microglial phagocytic activity and indoleamine 2,3 dioxygenase (IDO) activity (Dantzer et al., 2008; Jeon et al., 2010; Heneka et al., 2015). This microglial state presents higher rates of phagocytosis, especially near damaged neurons and neurotoxic aggregates, both *in vitro* and *in vivo* (Neher et al., 2011; Rajbhandari et al., 2014), which is considered to have a protective role against inflammatory microglia. However, although lipopolysaccharide (LPS) and neurotoxic aggregates are able to promote phagocytosis (Herber et al., 2004), chronic stimulation of these cells decreases their phagocytic capacity, causing a reduction in aggregate clearance, which may contribute to improving neurodegenerative processes (Mawuenyega et al., 2010; Krabbe et al., 2013; Hong et al., 2016). Furthermore, pro-inflammatory microglia are described as highly pro-oxidant (García-revilla et al., 2019), and could be considered as a neurotoxic phenomenon.

Microglia orchestrate a “defense and repair” mechanism once faced with a challenge. This inflammatory response *per se* is not an adverse process but is necessary to restore tissue homeostasis. Inflammatory processes work under tight control to ensure that microglia will be regulated toward a pro-resolutive state once their task has been completed. Considered a double-edged sword, this acute inflammatory response is a necessary mechanism against pathogens and damaged cells. However, if this response is prolonged, it may exacerbate neurodegeneration by placing pro-inflammatory microglial cells as relevant players acting as a hallmark of neurodegenerative diseases including Alzheimer’s disease, Parkinson’s disease, and amyotrophic lateral sclerosis (Shabab et al., 2017; Hickman et al., 2018).

## Microglial Priming

The concept of trained immunity, described mainly in peripheral innate immune cells, may explain microglial priming. It refers to the ability of these cells to develop and display memory for inflammatory and infectious challenges (Chapoval et al., 1998; Schroder et al., 2006; Perry and Holmes, 2014). Microglial priming is a long-lasting memory change of microglia, which occurs mainly after exposure of cells to inflammatory stimuli, such as LPS, inflammatory mediators, misfolded proteins, and neuronal fragments (Perry and Holmes, 2014; Haley et al., 2019). Primed microglia are more sensitive to potentially milder stimuli, where a second stimulus/hit leads to an exacerbated response compared to the first stimulus/hit. Basically, priming results in increased immune reactivity to secondary insult and also makes microglia more resistant to negative/regulatory feedback (Perry and Teeling, 2013; Perry and Holmes, 2014). Exaggerated inflammatory responses caused by microglia may impair homeostatic functions and lead to CNS damage, including impaired synaptic plasticity and neurodegeneration (Boje and Arora, 1992; Mizuno, 2015; Haruwaka et al., 2019; Therajaran et al., 2020; Vainchtein and Molofsky, 2020).

The idea of macrophage priming is well established *in vitro*. Treating these cells with interferon (IFN)-γ prior to a challenge with a Toll-like receptor (TLR) agonist enhances the response to the TLR agonist, probably due to activation of phosphoinositide 3-kinase (PI3K) and/or nuclear factor-kB (NF-kB; Schroder et al., 2006). *In vivo* studies show that preexposure to colony stimulating factor (CSF)-1 (intravenously and intraperitoneally) increases serum levels of IL-6 and TNF in response to a subsequent LPS challenge. In addition, isolated cells, such as peripheral blood leukocytes, spleen cells, and resident peritoneal cells from CSF-1-primed mice injected with LPS, release IL-6 constitutively (Chapoval et al., 1998). This process is accompanied by the long-term reprogramming of intracellular signaling and metabolic pathways and is fixed epigenetically (Lajqi et al., 2019).

The first evidence of microglial priming supported the idea that patients with chronic neurodegenerative diseases, such as Alzheimer’s disease, when affected by peripheral infections and systemic inflammation had greater consequences compared to healthy elderly individuals, such as greater chances of hospitalization, exacerbation of symptoms, and progression of neurodegeneration (Perry et al., 2007). Tahira et al. (2021) recently showed that Alzheimer’s disease is a risk factor for the severe form of COVID-19, also increasing the risk of mortality from coronavirus infection, regardless of age. Furthermore, MHC II is considered a marker of microglial priming in conditions such as aging and neurodegenerative diseases and is upregulated in the postmortem brain of patients with COVID-19 (Perry and Holmes, 2014; Matschke et al., 2020). Evidence for microglial priming has been shown in several models of neurodegenerative disorders, such as in transgenic mice with Alzheimer’s disease, in which LPS injection presented an increase in the inflammatory response (Sly et al., 2001). Furthermore, in a microglial priming model using a single intraperitoneal dose of methyl-4-phenyl-1,2,3,6-tetrahydropyridine (MPTP), and 4 days after the animals received a subtoxic dose of LPS, the animals showed an amplified inflammatory response, nigrostriatal dopaminergic degeneration, and an increase in protein expression levels of the components of the NLRP3 inflammasome and of NF-κB activity compared to animals that received only MPTP or LPS (Leem et al., 2021). Moreover, IL-1β injection intensifies cell degeneration in the substantia nigra and motor symptoms in a Parkinson’s disease model (Godoy et al., 2008). Thus, the additional stimulus in a microglial population due to a peripheral infection can aggravate inflammation and consequently cause greater harm to the patient. These findings demonstrate that microglial priming can occur in a pre-existing inflammation of the CNS, caused by a neurodegenerative disease, with a posterior peripheral infection. However, it is possible to suggest that this process is multimodal and therefore may be associated with the infectious process bilaterally.

## Could Prior Infections Influence The Development of Neurodegenerative Diseases?

After the 1918 influenza pandemic, epidemiological studies associated the outbreak of *Encephalitis lethargica* and Post-encephalitic Parkinsonism with the H1N1 pandemic (Ravenholt and Foege, 1982). A direct association between influenza and *encephalitis lethargica* was first reported in 1974, when viral antigens were found, using immunofluorescent staining, in the brain specimens of patients who had been diagnosed with *encephalitis lethargica* and Parkinsonism (Gamboa et al., 1974). However, these correlations were never proven and remain controversial (Mattock et al., 1988; Casals et al., 1998; Oxford, 2000; Henry et al., 2010; Vilensky et al., 2010; De Chiara et al., 2012; Dourmashkin et al., 2012).

Still, viral infections might provide the first stimulus that may lead to the subsequent development of neurodegenerative diseases (Estupinan et al., 2013). H1N1-infected mice given MPTP to induce Parkinson’s disease, present a decrease in the number of dopaminergic neurons in the substantia nigra pars compacta (SNpc) when compared to mice that were not previously infected with H1N1. Furthermore, animals previously vaccinated against H1N1 or treated with the antiviral drug oseltamivir carboxylate before MPTP exposure had similar numbers of dopaminergic neurons in the SNpc as control animals. These findings suggested that H1N1 infection alone was not able to cause Parkinsonism; however, it was responsible for priming the immune response in the brain, which could lead to Parkinson’s disease when another stimulus was added (Sadasivan et al., 2017). Thus, influenza led to the activation of the innate immune system in the brain, resulting in a later exacerbated response to the effects of a known Parkinsonian agent, MPTP.

Influenza infection and neurodegenerative disorders were also reported in studies by Ogata et al. (1997) where rodents exposed to the Japanese encephalitis virus presented neuronal loss, gliosis, and a decrease in the number of dopaminergic TH-positive neurons in the substantia nigra, all hallmarks of Parkinson’s disease. Infections with the neurotropic strain H5N1 in mice lead to neurodegeneration in the substantia nigra, especially in dopaminergic neurons (Jang et al., 2012), and even non-neurotropic strains, such as H1N1, also lead to brain inflammation and microgliosis in the hippocampus (Jurgens et al., 2012; Hosseini et al., 2018), despite the absence of virus in the brain (Sadasivan et al., 2015), indicating that both neurotropic and non-neurotropic influenza can lead to neurodegeneration.

The connection between viral infections and neurodegenerative disease is not limited to influenza; other viral infections may also result in neurodegeneration. Human herpes virus-6 (HHV-6) expression (mRNA and protein), was detected in a periventricular multiple sclerosis lesion, specifically in oligodendrocytes (Leibovitch and Jacobson, 2014). Furthermore, HHV-6 is associated with the development of Alzheimer’s disease. Readhead et al. (2018) using a multiscale analysis in postmortem brain tissue from patients with Alzheimer’s disease, observed a relationship between the viral abundance of HHV-6 and 7 and genes related to amyloid precursor protein, an important feature of Alzheimer’s disease. In addition, recent studies have shown the relationship between Epstein-Barr infection (EBV) and multiple sclerosis (Bjornevik et al., 2022).

Viral infections are associated with neurodegeneration hallmarks. Parasite infections have also been associated with the development of neurodegenerative disorders. Mice infected with *Toxoplasma gondii* associated with the administration of subdoses of Aβ peptide presented significant impairments in learning and memory functions and increased IL-1β, TNF-α, IFN-γ, and inducible nitric oxide synthase (iNOS) mRNA levels, similar to the Alzheimer’s disease group, which received high doses of Aβ but were not infected (Mahmoudvand et al., 2016). In recent decades, many studies have investigated the mechanisms related to long-term cognitive and behavioral sequelae resulting from malaria infection. Microglial changes are observed in experimental models of malaria and in *postmortem* brains of patients with malaria (Janota and Doshi, 1979; Schluesener et al., 1998; Talavera-López et al., 2018). Microglial human cells phagocytize extracellular vesicles derived from red blood cells infected with *Plasmodium falciparum*, the main parasite that causes cerebral malaria in humans, resulting in morphological changes including cytoplasmic granulations, formation of numerous pseudopods, process retraction and cell body swelling (Mbagwu et al., 2020). In an experimental model of cerebral malaria, magnetic resonance imaging revealed the time course of rupture of the BBB, an event essential for the development of this cerebral malaria, beginning in the olfactory bulb and spreading along the rostral migratory flow accompanied by a specific route of microglial changes related to the pro-inflammatory state (Hoffmann et al., 2016). In experimental malaria models, cognitive dysfunction has been correlated with the pro-inflammatory microglial state (Desruisseaux et al., 2008; Guha et al., 2014; Lacerda-Queiroz et al., 2015; Souza et al., 2018; Andoh and Gyan, 2021). Microglial transcriptomic analysis revealed that in the acute phase of experimental malaria infection, where the brain is already severely affected, there is increased expression of genes related to immune responses (Capuccini et al., 2016; Talavera-López et al., 2018). BV-2 cells, a microglial cell line, stimulated with hemozoin, a molecule resulting from plasmodium metabolism, induced an increase in the production of TNFα, IL-6, IL-1β, and NO (Velagapudi et al., 2019). Furthermore, minocycline treatment, a drug that has been described to regulate microglial functions, including inhibition of the pro-inflammatory state and restoring phagocytic functions, prevented the development of cerebral malaria and conferred neuroprotection in infected mice (Markovic et al., 2011; Kobayashi et al., 2013; Hu et al., 2014; Apoorv and Babu, 2017; Bassett et al., 2021; Paolicelli et al., 2022). Taking into account the concept of microglial priming, more studies are needed to evaluate the involvement of microglia in the pathogenesis and development of neurodegenerative diseases in malaria survivor patients, as this disease leaves long-term neurological sequelae.

Bacterial infections can also promote neural damage and changes in the microglial response. Mice previously challenged with *Salmonella typhimurium* presented a robust inflammatory response to low doses of injection of LPS into the brain, but in previously uninfected mice these low doses did not evoke or evoke a small inflammatory response (Püntener et al., 2012). Furthermore, LPS injection promoted axonal injury in an experimental model of multiple sclerosis, a key contributor to the progression of disability in multiple sclerosis (Moreno et al., 2011). The LPS challenge was performed in the reemission phase of the disease, causing systemic inflammation that correlates with pro-inflammatory microglia and axonal damage, suggesting that systemic infections can contribute to the aggravation of neurodegenerative diseases. *Porphyromonas gingivalis* infection in the transgenic mouse model of Alzheimer’s disease (APP-Tg) increased the deposition of Aβ and the levels of inflammatory cytokines in the brain. They also observed that microglial cell cultures that were previously exposed to Aβ oligomers and then exposed to *Porphyromonas gingivalis* endotoxin presented an increase in TNF-α and IL-1β production (Ishida et al., 2017). Septic mice have been reported to be more susceptible to Aβ oligomers, and this could be due to a long-lasting trained innate immunological memory (De Sousa et al., 2021). Microglial cells from surviving septic mice appear to be more responsive and are more prone to shift to a pro-inflammatory profile, when exposed to smaller amounts of Aβ when compared to control mice, leading to an increase in synapses phagocytosis in the hippocampus. Pharmacological blockade of brain phagocytic cells or microglial depletion, using minocycline and CSF-1 receptor inhibitor (PLX3397), respectively, prevented AβO-induced cognitive dysfunction in surviving septic mice (De Sousa et al., 2021). Furthermore, using a transgenic animal model for Alzheimer’s disease (APP/PS1–21 transgenic mouse) that underwent a septic event through cecal ligation and puncture (CLP) surgery, the animals presented increased fibrillar amyloid plaque formation in the hippocampus, in addition to the slight changes found in the microglia (Basak et al., 2021). Together, these results suggest that sepsis could be a potential factor in increasing dementia and may contribute to the mechanisms involved in exacerbated amyloid plaque deposition.

Furthermore, some evidence suggests that inflammation resulting from an uncontrolled microglial response may precede the development of diseases known as tauopathies. The Tau protein is a soluble protein associated with microtubules that is expressed primarily by neurons located in the cytoplasm and axons (Gorath et al., 2001; Wang et al., 2013). These proteins have been shown to be involved in the pathology of several neurodegenerative diseases, including Alzheimer’s disease. Aggregated and hyperphosphorylated tau proteins form the core of neurofibrillary tangles, which are one of the pathological hallmarks of Alzheimer’s disease. The mechanisms involved in these pathologies are still not completely understood, but exosomes may be an important link between tau propagation and the pro-inflammatory microglial state (Gao et al., 2018; Španić et al., 2019). Reducing the number of microglial cells and inhibiting exosome synthesis reduces the spread of tau proteins (Asai et al., 2015); thus, serum levels of tau protein are useful to support findings of acute neuronal damage, including acute ischemic stroke, traumatic brain injury, intracranial hemorrhage, epilepsy, and cardiac arrest (Bitsch et al., 2002; Palmio et al., 2009; Hu et al., 2012; Randall et al., 2013; Mattsson et al., 2017; Tang et al., 2019; Nakada et al., 2020). In patients with sepsis, serum tau protein levels were significantly higher in the group that did not survive compared to the surviving individuals; therefore, it may be useful as a mortality predictor in patients with severe sepsis (Zhao et al., 2019). Furthermore, rats that received a single dose of LPS showed hippocampal deposition of intracellular phosphorylated tau protein (Kirk et al., 2019). More studies are needed to understand the association of tau protein with neurodegenerative diseases; however, these findings may help explain the higher rate of dementia observed in longitudinal studies of septic survivors (Chou et al., 2017).

## Microglial Priming Induced in Brain Inflammation Could Be A Key Factor Between Prior Infections and Neurogenerative Diseases

Brain inflammation can occur as a result of direct injury to the CNS, such as infection, trauma, or neurodegenerative diseases, but also may be a consequence of systemic inflammation caused by infections (Young, 2013) of which microglial cells are key players. There are several routes of communication between the periphery and the brain, but we can highlight three major pathways. First, afferent nerves, such as vagal nerves in adnominal infections and trigeminal nerves in orolingual infections (Bluthé et al., 1996; Ek et al., 1998). Second, inflammatory mediators present in the circulation communicate directly with circumventricular organs, which do not have an intact BBB; then, this signaling is spread by microglia into the brain parenchyma (Quan et al., 1998). Third, pro-inflammatory mediators or microbial products can interact directly with cells in the brain endothelium, signaling directly through the BBB and with perivascular macrophages (Vitkovic et al., 2000). All these routes will allow the brain to recognize the peripheral inflammatory state and then glial cells, mainly microglia, will respond by releasing pro-inflammatory molecules.

Once in the brain, cytokines lead to several changes in the states of glial cells (microglia and astrocytes), neurotoxic mechanisms, and modulate neurotransmitter metabolism (Heneka et al., 2015; DiSabato et al., 2016). This inflammatory scenario plays an important role in the progression of neurodegenerative diseases. Seminal articles in this area described highly reactive microglia, evaluated by the expression of the human leukocyte antigen DR isotype (HLA-DR) and other immune cells, such as T cells, in the brains of patients with Alzheimer’s and Parkinson’s disease (McGeer et al., 1987, 1988; Itagaki et al., 1988). These studies laid the foundation for considering brain inflammation as a potential factor involved in the onset and/or development of these pathologies.

Neurodegenerative diseases are not easy to diagnose due to the heterogeneity of pathological biomarkers, most are only identifiable by postmortem examination. But there are some hallmarks associated with loss of neurons and synapses, gliosis, and vascular abnormalities in specific regions of the brain, and in inflammation (Villoslada et al., 2020). The persistent inflammatory state found in neurodegenerative diseases is detrimental to microglia and other glial cells, as this continuous state increases ROS and NO production. In a vicious cycle, inflammation eventually can lead to neurodegeneration resulting in protein aggregation, dysfunctional cells, and neuronal death (Arcuri et al., 2017; Marttinen et al., 2018). In Parkinson’s disease, pro-inflammatory microglia phagocytose injured neurons and induce the release of inflammatory mediators, including ROS, NO, and IL-6; These mediators will contribute to astrogliosis that exacerbates inflammation and increases the phagocytosis of neuronal debris mediated by microglia cells. Furthermore, the aggregation of alpha-synuclein itself accelerates this process by inducing IL-1β releasing *via* TLR signaling (Hickman et al., 2018; Marttinen et al., 2018; Subhramanyam et al., 2019). A similar process related to Aβ aggregation also occurs in Alzheimer’s disease (Marttinen et al., 2018). This evidence suggests that microglia cells contribute to the initiation and amplification of the neurodegeneration process.

**Figure 1:**
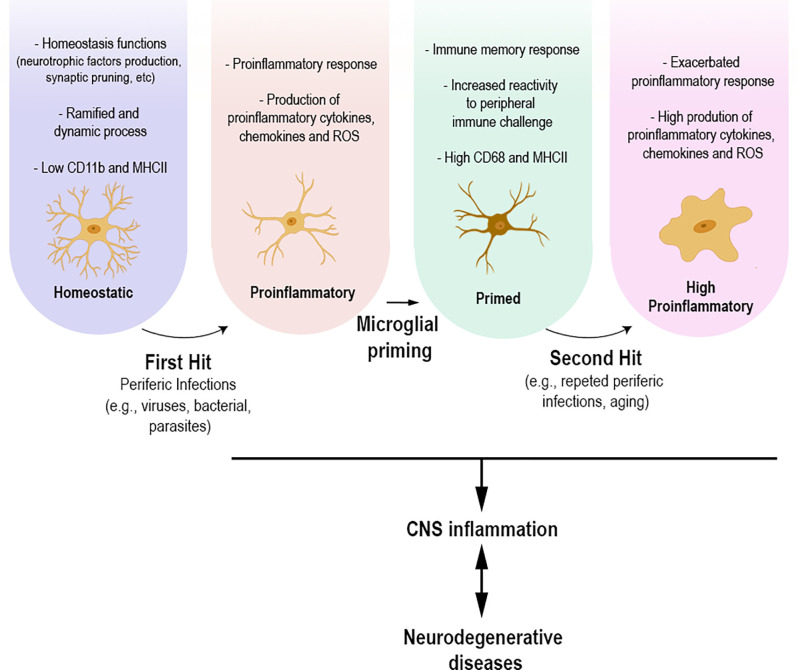
Under physiological conditions, homeostatic microglial cells participate in various homeostasis functions, production of neurotrophic factors, and synaptic pruning. Their morphology presents ramified and dynamic cellular processes with high motility. Homeostatic microglia express low levels of CD11b and MHCII. Infections that occur throughout life, whether caused by viruses, parasites, or bacteria, can lead to microglial morphological and functional changes towards a proinflammatory state. The proinflammatory response leads to functional and morphological changes, including the upregulation of specific molecules and increased production of proinflammatory mediators, including cytokines, chemokines, and reactive oxygen species. Microglial cells present a branch architecture with thicker processes. This process can result in microglial priming, making these cells more responsive to an upcoming insult, causing an, even more, exacerbated inflammatory response. The different states of microglia are directly related to the inflammation process, which in turn might be related to the development of neurodegenerative diseases.

The common point between infectious diseases and neurodegenerative disorders appears to be the inflammatory process, which involves a microglial response. Experimental evidence is still preliminary; however, it allows us to hypothesize that brain inflammation caused by peripheral infections—or repeated exposure to infections throughout life—may be/act as an initial microglial priming event, first hit, and subsequent neuroinflammatory stimuli, second hit, may promote an exacerbated microglial pro-inflammatory response, causing a series of biological events related to the development of neurodegenerative diseases (Figure 1). Finally, infections that occur in early life also highlight the role of microglial priming. Infections at this period of life are described leaving long-term sequelae, such as cognitive deficits, in adulthood (Bilbo et al., 2005; Ratnayake et al., 2013; Li et al., 2014; Han et al., 2017; Osborne et al., 2017; Granja et al., 2021). Experimental models show that microglial priming alone does not lead to these deficits, as studies emphasize the importance of a second stimulus/hit for the development of cognitive sequelae (Bilbo et al., 2005; Bilbo, 2010; Li et al., 2014; Osborne et al., 2017). In addition, the role of infections in early life and their role as risk factors for the development of neurodegenerative diseases have been discussed (Miller and O’Callaghan, 2008).

The impact of systemic inflammation on the development of neurodegenerative diseases has been extensively explored. LPS administration, in different experimental models, has been described to increase the deposition of Aβ protein, cognitive deficits, phosphorylated tau protein, and decrease the level of dopaminergic neurons that are hallmarks of Alzheimer’s and Parkinson’s disease, respectively (Lee et al., 2008; Kahn et al., 2012; Kiyofuji et al., 2015; Kirk et al., 2019; Tejera et al., 2019; Wang et al., 2020). Not only is LPS injection related to the development of neurodegenerative diseases, systemic delivery of TNF-α leads to disease behavior, cognitive deficit, increase in tau expression, and IBA-1 staining in the mouse hippocampus (Hennessy et al., 2017). Furthermore, systemic inflammatory challenge in the prenatal phase, at the end of gestation, with viral mimic, polyriboinosinic-polyribocytidyl acid (PolyI:C) predisposes healthy mice to develop a pathology similar to Alzheimer’s disease (Krstic et al., 2012). Animals showed high levels of IL-1β, IL-1α, and IL-6 in plasma and the brain in the early stages and during aging (3–15 months of life). Microglial cells with altered activity were observed by immunostaining with CD68, a marker of phagocytosis and antigen presentation, in the CA1 region of the hippocampus (Krstic et al., 2012). Furthermore, prenatal exposure to PolyI:C resulted in a significant age-dependent increase in the amount of amyloid precursor protein (APP) in the hippocampus and altered Tau phosphorylation and significant impairment of working memory in old-age mice, evaluated by the Y-maze test (Krstic et al., 2012). Although a single prenatal immune challenge was sufficient to generate a chronic inflammatory state of the CNS in animals and increase the vulnerability of the brain to the development of neurological diseases, the study even showed that a second immune challenge in adulthood exacerbated this state, including increased APP deposition, Tau protein aggregation, and microglial profile modification (Krstic et al., 2012). These results show that it is possible that early or repeated exposure to inflammation is an initiating event for microglia that may lead to long-term sequelae, including an increased predisposition to the development of neurodegenerative diseases.

Microglial priming is directly related to the exacerbation of the inflammatory response and, consequently, to increased brain inflammation and subsequent damage. Mice challenged with LPS 12 weeks after inoculation with ME7 prion, a neurodegenerative experimental model used to study brain inflammation (Chouhan et al., 2017), demonstrated exacerbated neuronal death, behavioral deficits, increased IL-1β, TNF-α, and IFN-β levels in the brain, and increased microglial IL-1β (Cunningham et al., 2009; Murray et al., 2011). Priming BV-2 microglial cells with IFN-γ substantially increased the production of ROS after microglial stimulation with ATP (Spencer et al., 2016). IFNγ-induced priming by promoting upregulation of the NADPH oxidase NOX2 subunit and reducing intracellular glutathione levels (Spencer et al., 2016). Oxidative stress and subsequently mitochondrial damage are one of the main causes of neuronal damage in several brain disorders including Alzheimer’s and Parkinson’s disease and are mainly caused by pro-inflammatory microglia due to excessive ROS production mediated by NADPH oxidase (Simpson and Oliver, 2020). Considering that infectious diseases lead to the synthesis of several pro-inflammatory cytokines such as IFN-γ, mitochondrial oxidative damage *via* ROS-induced by pro-inflammatory cytokines could be one of the mechanisms by which infectious diseases lead to neurodegenerative conditions (Brown et al., 1999; Romero et al., 2010; Sturge and Yarovinsky, 2014; Kyuwa and Sugiura, 2020).

Thus, repeated infectious processes can act as a second hit and trigger a response in the primed microglia. However, it is important to emphasize that the aging process itself can be considered a second hit. It was shown that early postnatal infection of rats with LPS combined with the aging process resulted in less successful cognitive aging in these animals (Bilbo, 2010). Aging is a risk factor for the development of many neurodegenerative diseases because the natural aging process includes functional and structural changes within the brain (Bennett et al., 1996; Reeve et al., 2014; Maniega et al., 2015; Chen et al., 2020). Among these changes is immune system dysfunction, which generates a low-grade chronic pro-inflammatory condition called inflammageing (Franceschi et al., 2018). Furthermore, aging-related microglia in the aged brain and the pro-inflammatory microglial state show decreased motility, metabolic and immune changes, such as increased constitutive production of pro-inflammatory cytokines (e.g., TNF-α, IL-1β, and IL-6; Sierra et al., 2007; Hefendehl et al., 2014; Marschallinger et al., 2020; Schiess et al., 2020; Shaerzadeh et al., 2020). RNAseq analysis of microglia from elderly mice showed that the pathway of genes related to phagosome maturation and NO and ROS production was dysregulated, producing an impairment of essential microglial functions (Marschallinger et al., 2020). Although aging is not a disease, it can lead to nonbeneficial changes in the physiological role of microglia and may be a microglial priming factor. Therefore, the dysfunctional role of microglia and the chronic inflammatory microenvironment may be associated with neurodegeneration. Peripheral LPS injection into aged mice enhanced cortical microglial response and increased IL-1β and IDO mRNA levels in microglia isolated from aged mice compared to adult mice (Wynne et al., 2009). These findings suggest that an inflammatory condition, caused by aging, before a second stimulus contributes to exacerbating a subsequent response.

CX3CL1-CX3CR1 signaling is essential for microglia-neuron communication and may play an important role in the progression of neurodegenerative diseases. In the hippocampus of elderly rats, CX3CL1 expression is reduced and this is correlated with changes in microglia, including increased expression of CD40 mRNA and MHC II and IL-1β expression (Lyons et al., 2009). In adult mice, microglia can restore CX3CR1 levels 24 h after LPS challenge, whereas CX3CR1 was still significantly reduced in the microglia of aged mice (Wynne et al., 2010). In Alzheimer’s disease, CX3CL1 expression is reduced in the main areas where the pathological changes occur and its expression levels reflect the progression of the disease (Duan et al., 2008). CX3CL1 is also decreased in the cerebrospinal fluid of patients with Alzheimer’s disease, and patients with mild to moderate Alzheimer’s disease have significantly higher plasma CX3CL1 levels compared to patients with the severe form of the disease (Kim et al., 2008; Perea et al., 2018b). In APPPS1 mice, an Alzheimer’s disease study model, CX3CR1 knockout showed a reduction in Aβ deposition and fewer CD68 positive microglial cells (Lee et al., 2010). Furthermore, CX3CR1 deficiency reduced the number of microglia surrounding Aβ deposits in a dose-dependent manner of with levels of CX3CR1 gene expression (Lee et al., 2010). The impairment of CX3CL1-CX3CR1 signaling, which mainly impacts the functional signaling of microglia, seems to be a central point between inflammageing and the development of neurodegenerative diseases.

## Conclusion

The relationship between infection and neurodegenerative diseases could be subtle and may rely on microglial priming. Infections, even non-neurotropic ones, may lead to an inflammatory condition in the brain mainly mediated by the pro-inflammatory microglial state. The inflammatory response caused by infections has been associated with microglial priming events that may induce changes in their state and function. Despite priming events that prepare microglial cells for subsequent immune challenges to promote repair and homeostasis, microglia are long-lived cells that are constantly exposed to infections during an individual’s lifetime, and this also may not be beneficial, as it may contribute indirectly to neurodegenerative disorders.

## Author Contributions

All authors made substantial contributions and participated in drafting the article and revising it critically. All authors contributed to the article and approved the submitted version.

## Conflict of Interest

The authors declare that the research was conducted in the absence of any commercial or financial relationships that could be construed as a potential conflict of interest.

## Publisher’s Note

All claims expressed in this article are solely those of the authors and do not necessarily represent those of their affiliated organizations, or those of the publisher, the editors and the reviewers. Any product that may be evaluated in this article, or claim that may be made by its manufacturer, is not guaranteed or endorsed by the publisher.

## References

[B1] Andoh N. E. Gyan B. A. (2021). The potential roles of glial cells in the neuropathogenesis of cerebral malaria. Front. Cell. Infect. Microbiol. 11:741370. 10.3389/fcimb.2021.741370 34692564 PMC8529055

[B2] Apoorv T. S. Babu P. P. (2017). Minocycline prevents cerebral malaria, confers neuroprotection and increases survivability of mice during Plasmodium berghei ANKA infection. Cytokine 90, 113–123. 10.1016/j.cyto.2016.11.001 27865203

[B3] Arcuri C. Mecca C. Bianchi R. Giambanco I. Donato R. (2017). The pathophysiological role of microglia in dynamic surveillance, phagocytosis and structural remodeling of the developing CNS. Front. Mol. Neurosci. 10:191. 10.3389/fnmol.2017.00191 28674485 PMC5474494

[B4] Arnò B. Grassivaro F. Rossi C. Bergamaschi A. Castiglioni V. Furlan R.. (2014). Neural progenitor cells orchestrate microglia migration and positioning into the developing cortex. Nat. Commun. 5:5611. 10.1038/ncomms6611 25425146

[B5] Asai H. Ikezu S. Tsunoda S. Medalla M. Luebke J. Haydar T.. (2015). Depletion of microglia and inhibition of exosome synthesis halt tau propagation. Nat. Neurosci. 18, 1584–1593. 10.1038/nn.4132 26436904 PMC4694577

[B6] Barbosa-Silva M. C. Lima M. N. Battaglini D. Robba C. Pelosi P. Rocco P. R. M.. (2021). Infectious disease-associated encephalopathies. Crit. Care 25:236. 10.1186/s13054-021-03659-6 34229735 PMC8259088

[B7] Barbosa-Silva M. C. Santos L. E. Rangel B. (2018). The impact of non-neurotropic influenza strains on the brain: a role for microglial priming? J. Neurosci. 38, 7758–7760. 10.1523/JNEUROSCI.1368-18.2018 30185538 PMC6596083

[B8] Basak J. M. Ferreiro A. Cohen L. S. Sheehan P. W. Nadarajah C. J. Kanan M. F.. (2021). Bacterial sepsis increases hippocampal fibrillar amyloid plaque load and neuroinflammation in a mouse model of Alzheimer’s disease. Neurobiol. Dis. 152:105292. 10.1016/j.nbd.2021.105292 33556539 PMC8057119

[B9] Bassett B. Subramaniyam S. Fan Y. Varney S. Pan H. Carneiro A. M. D.. (2021). Minocycline alleviates depression-like symptoms by rescuing decrease in neurogenesis in dorsal hippocampus via blocking microglia activation/phagocytosis. Brain. Behav. Immun. 91, 519–530. 10.1016/j.bbi.2020.11.009 33176182

[B10] Bellucci A. Westwood A. J. Ingram E. Casamenti F. Goedert M. Spillantini M. G. (2004). Induction of inflammatory mediators and microglial activation in mice transgenic for mutant human P301S tau protein. Am. J. Pathol. 165, 1643–1652. 10.1016/S0002-9440(10)63421-9 15509534 PMC1618683

[B11] Bennett D. A. Beckett L. A. Murray A. M. Shannon K. M. Goetz C. G. Pilgrim D. M.. (1996). Prevalence of parkinsonian signs and associated mortality in a community population of older people. N. Engl. J. Med. 334, 71–76. 10.1056/NEJM199601113340202 8531961

[B12] Bilbo S. D. (2010). Early-life infection is a vulnerability factor for aging-related glial alterations and cognitive decline. Neurobiol. Learn. Mem. 94, 57–64. 10.1016/j.nlm.2010.04.001 20388544 PMC2881165

[B13] Bilbo S. D. Levkoff L. H. Mahoney J. H. Watkins L. R. Rudy J. W. Maier S. F. (2005). Neonatal infection induces memory impairments following an immune challenge in adulthood. Behav. Neurosci. 119, 293–301. 10.1037/0735-7044.119.1.293 15727533

[B14] Bitsch A. Horn C. Kemmling Y. Seipelt M. Hellenbrand U. Stiefel M.. (2002). Serum tau protein level as a marker of axonal damage in acute ischemic stroke. Eur. Neurol. 47, 45–51. 10.1159/000047946 11803192

[B15] Bjornevik K. Cortese M. Healy B. C. Kuhle J. Mina M. J. Leng Y.. (2022). Longitudinal analysis reveals high prevalence of Epstein-Barr virus associated with multiple sclerosis. Science 375, 296–301. 10.1126/science.abj8222 35025605

[B16] Bluthé R. M. Michaud B. Kelley K. W. Dantzer R. (1996). Vagotomy attenuates behavioural effects of interleukin-1 injected peripherally but not centrally. Neuroreport 7, 1485–1488. 10.1097/00001756-199606170-00008 8856703

[B17] Boje K. M. Arora P. K. (1992). Microglial-produced nitric oxide and reactive nitrogen oxides mediate neuronal cell death. Brain Res. 587, 250–256. 10.1016/0006-8993(92)91004-x 1381982

[B18] Brown H. Turner G. Rogerson S. Tembo M. Mwenechanya J. Molyneux M.. (1999). Cytokine expression in the brain in human cerebral malaria. J. Infect. Dis. 180, 1742–1746. 10.1086/315078 10515846

[B19] Capuccini B. Lin J. Talavera-López C. Khan S. M. Sodenkamp J. Spaccapelo R.. (2016). Transcriptomic profiling of microglia reveals signatures of cell activation and immune response, during experimental cerebral malaria. Sci. Rep. 6:39258. 10.1038/srep39258 27991544 PMC5171943

[B78] Casals J. Elizan T. S. Yahr M. D. (1998). Postencephalitic parkinsonism-a review. J. Neural Transm. (Vienna) 105, 645–676. 10.1007/s007020050086 9826109

[B20] Chapoval A. I. Kamdar S. J. Kremlev S. G. Evans R. (1998). CSF-1 (M-CSF) differentially sensitizes mononuclear phagocyte subpopulations to endotoxin *in vivo*: a potential pathway that regulates the severity of gram-negative infections. J. Leukoc. Biol. 63, 245–252. 10.1002/jlb.63.2.245 9468283

[B21] Chen M. B. Yang A. C. Yousef H. Lee D. Chen W. Schaum N.. (2020). Brain endothelial cells are exquisite sensors of age-related circulatory cues. Cell Rep. 30, 4418–4432.e4. 10.1016/j.celrep.2020.03.012 32234477 PMC7292569

[B22] Chou C. H. Lee J. T. Lin C. C. Sung Y. F. Lin C. C. Muo C. H.. (2017). Septicemia is associated with increased risk for dementia: a population-based longitudinal study. Oncotarget 8, 84300–84308. 10.18632/oncotarget.20899 29137424 PMC5663596

[B23] Chouhan J. K. Fowler S. B. Webster C. I. Teeling J. L. (2017). The ME7 prion model of neurodegeneration as a tool to understand target neuroinflammation in Alzheimer’s disease. Drug Discov. Today Dis. Model. 25–26, 45–52. 10.1016/j.ddmod.2018.10.004

[B24] Colton C. A. Wilcock D. M. (2010). Assessing activation states in microglia. CNS Neurol. Disord. Drug Targets 9, 174–191. 10.2174/187152710791012053 20205642

[B25] Cunningham C. Campion S. Lunnon K. Murray C. L. Woods J. F. C. Deacon R. M. J.. (2009). Systemic inflammation induces acute behavioral and cognitive changes and accelerates neurodegenerative disease. Biol. Psychiatry 65, 304–312. 10.1016/j.biopsych.2008.07.024 18801476 PMC2633437

[B26] Czirr E. Castello N. A. Mosher K. I. Castellano J. M. Hinkson I. V. Lucin K. M.. (2017). Microglial complement receptor 3 regulates brain Aβ levels through secreted proteolytic activity. J. Exp. Med. 214, 1081–1092. 10.1084/jem.20162011 28298456 PMC5379986

[B27] d’Errico P. Ziegler-Waldkirch S. Aires V. Hoffmann P. Mezö C. Erny D.. (2022). Microglia contribute to the propagation of Aβ into unaffected brain tissue. Nat. Neurosci. 25, 20–25. 10.1212/WNL.0000000000200614 34811521 PMC8737330

[B28] Dantzer R. Connor J. C. O. Freund G. G. Johnson R. W. Kelley K. W. (2008). From inflammation to sickness and depression: when the immune system subjugates the brain. Nat. Rev. Neurosci. 9, 46–56. 10.1038/nrn2297 18073775 PMC2919277

[B29] Davalos D. Grutzendler J. Yang G. Kim J. V. Zuo Y. Jung S.. (2005). ATP mediates rapid microglial response to local brain injury *in vivo*. Nat. Neurosci. 8, 752–758. 10.1038/nn1472 15895084

[B30] Davis E. J. Foster T. D. Thomas W. E. (1994). Cellular forms and functions of brain microglia. Brain Res. Bull. 34, 73–78. 10.1016/0361-9230(94)90189-9 8193937

[B33] De S. Van Deren D. Peden E. Hockin M. Boulet A. Titen S.. (2018). Two distinct ontogenies confer heterogeneity to mouse brain microglia. Development 145:dev152306. 10.1242/dev.152306 29973370 PMC6053660

[B31] De Chiara G. Marcocci M. E. Sgarbanti R. Civitelli L. Ripoli C. Piacentini R.. (2012). Infectious agents and neurodegeneration. Mol. Neurobiol. 46, 614–638. 10.1007/s12035-012-8320-7 22899188 PMC3496540

[B32] De Sousa V. L. Araújo S. B. Antonio L. M. Silva-Queiroz M. Colodeti L. C. Soares C.. (2021). Innate immune memory mediates increased susceptibility to Alzheimer’s disease-like pathology in sepsis surviving mice. Brain. Behav. Immun. 95, 287–298. 10.1016/j.bbi.2021.04.001 33838250

[B34] Desruisseaux M. S. Gulinello M. Smith D. N. Lee S. H. C. Tsuji M. Weiss L. M.. (2008). Cognitive dysfunction in mice infected with *Plasmodium berghei* strain ANKA. J. Infect. Dis. 197, 1621–1627. 10.1086/587908 18419550 PMC2692506

[B35] DiSabato D. Quan N. Godbout J. P. (2016). Neuroinflammation: the devil is in the details. J. Neurochem. 139, 136–153. 10.1111/jnc.13607 26990767 PMC5025335

[B36] Dourmashkin R. R. Dunn G. Castano V. McCall S. A. (2012). Evidence for an enterovirus as the cause of encephalitis lethargica. BMC Infect. Dis. 12:136. 10.1186/1471-2334-12-136 22715890 PMC3448500

[B37] Duan R. S. Yang X. Chen Z. G. Lu M. O. Morris C. Winblad B.. (2008). Decreased fractalkine and increased IP-10 expression in aged brain of APPswe transgenic mice. Neurochem. Res. 33, 1085–1089. 10.1007/s11064-007-9554-z 18095157

[B38] Ehlers M. R. W. (2000). CR3: a general purpose adhesion-recognition receptor essential for innate immunity. Microbes Infect. 2, 289–294. 10.1016/s1286-4579(00)00299-9 10758405

[B39] Ek M. Kurosawa M. Lundeberg T. Ericsson A. (1998). Activation of vagal afferents after intravenous injection of interleukin-1β: role of endogenous prostaglandins. J. Neurosci. 18, 9471–9479. 10.1523/JNEUROSCI.18-22-09471.1998 9801384 PMC6792875

[B40] Estupinan D. Nathoo S. Okun M. S. (2013). The demise of poskanzer and schwab’s influenza theory on the pathogenesis of Parkinson’s disease. Parkinsons Dis. 2013:167843. 10.1155/2013/167843 23853734 PMC3693163

[B41] Fernández-Arjona M. D. M. Grondona J. M. Granados-Durán P. Fernández-Llebrez P. López-Ávalos M. D. (2017). Microglia morphological categorization in a rat model of neuroinflammation by hierarchical cluster and principal components analysis. Front. Cell. Neurosci. 11:235. 10.3389/fncel.2017.00235 28848398 PMC5550745

[B42] Franceschi C. Garagnani P. Parini P. Giuliani C. Santoro A. (2018). Inflammaging: a new immune-metabolic viewpoint for age-related diseases. Nat. Rev. Endocrinol. 14, 576–590. 10.1038/s41574-018-0059-4 30046148

[B43] Franco-Bocanegra D. K. Gourari Y. McAuley C. Chatelet D. S. Johnston D. A. Nicoll J. A. R.. (2021). Microglial morphology in Alzheimer’s disease and after Aβ immunotherapy. Sci. Rep. 11:15955. 10.1038/s41598-021-95535-0 34354209 PMC8342480

[B44] Frank M. G. Wieseler-Frank J. L. Watkins L. R. Maier S. F. (2006). Rapid isolation of highly enriched and quiescent microglia from adult rat hippocampus: immunophenotypic and functional characteristics. J. Neurosci. Methods 151, 121–130. 10.1016/j.jneumeth.2005.06.026 16125247

[B45] Friedman B. A. Srinivasan K. Ayalon G. Meilandt W. J. Lin H. Huntley M. A.. (2018). Diverse brain myeloid expression profiles reveal distinct microglial activation states and aspects of Alzheimer’s disease not evident in mouse models. Cell Rep. 22, 832–847. 10.1016/j.celrep.2017.12.066 29346778

[B46] Furube E. Kawai S. Inagaki H. Takagi S. Miyata S. (2018). Brain region-dependent heterogeneity and dose-dependent difference in transient microglia population increase during lipopolysaccharide-induced inflammation. Sci. Rep. 8:2203. 10.1038/s41598-018-20643-3 29396567 PMC5797160

[B47] Gamboa E. T. Wolf A. Yahr M. D. Harter D. H. Duffy P. E. Barden H.. (1974). Influenza virus antigen in postencephalitic Parkinsonism brain: detection by immunofluorescence. Arch. Neurol. 31, 228–232. 10.1001/archneur.1974.00490400042003 4370102

[B48] Gao Y.-L. Wang N. Sun F. R. Cao X.-P. Zhang W. Yu J.-T. (2018). Tau in neurodegenerative disease. Ann. Transl. Med. 6:175. 10.21037/atm.2018.04.23 29951497 PMC5994507

[B49] García-revilla J. Alonso-bellido I. M. Burguillos M. A. Herrera A. J. Espinosa-oliva A. M. Ruiz R.. (2019). Reformulating pro-oxidant microglia in neurodegeneration. J. Clin. Med. 8:1719. 10.3390/jcm8101719 31627485 PMC6832973

[B50] Godoy M. C. P. Tarelli R. Ferrari C. C. Sarchi M. I. Pitossi F. J. (2008). Central and systemic IL-1 exacerbates neurodegeneration and motor symptoms in a model of Parkinson’s disease. Brain 131, 1880–1894. 10.1093/brain/awn101 18504291 PMC2442423

[B51] Gorath M. Stahnke T. Mronga T. Goldbaum O. Richter-Landsberg C. (2001). Developmental changes of tau protein and mRNA in cultured rat brain oligodendrocytes. Glia 36, 89–101. 10.1002/glia.1098 11571787

[B52] Granja M. G. Alves L. P. Leardini-Tristão M. Saul M. E. Bortoni L. C. de Moraes F. M.. (2021). Inflammatory, synaptic, motor and behavioral alterations induced by gestational sepsis on the offspring at different stages of life. J. Neuroinflammation 18:60. 10.1186/s12974-021-02106-1 33632243 PMC7905683

[B53] Guha S. K. Tillu R. Sood A. Patgaonkar M. Nanavaty I. N. Sengupta A.. (2014). Single episode of mild murine malaria induces neuroinflammation, alters microglial profile, impairs adult neurogenesis and causes deficits in social and anxiety-like behavior. Brain. Behav. Immun. 42, 123–137. 10.1016/j.bbi.2014.06.009 24953429

[B54] Haley M. J. Brough D. Quintin J. Allan S. M. (2019). Microglial priming as trained immunity in the brain. Neuroscience 405, 47–54. 10.1016/j.neuroscience.2017.12.039 29292078

[B55] Han Q. Lin Q. Huang P. Chen M. Hu X. Fu H.. (2017). Microglia-derived IL-1β contributes to axon development disorders and synaptic deficit through p38-MAPK signal pathway in septic neonatal rats. J. Neuroinflammation 14:52. 10.1186/s12974-017-0805-x 28288671 PMC5348817

[B56] Haruwaka K. Ikegami A. Tachibana Y. Ohno N. Konishi H. Hashimoto A.. (2019). Dual microglia effects on blood brain barrier permeability induced by systemic inflammation. Nat. Commun. 10:5816. 10.1038/s41467-019-13812-z 31862977 PMC6925219

[B57] Hefendehl J. K. Neher J. J. Sühs R. B. Kohsaka S. Skodras A. Jucker M. (2014). Homeostatic and injury-induced microglia behavior in the aging brain. Aging Cell 13, 60–69. 10.1111/acel.12149 23953759 PMC4326865

[B58] Heneka M. T. Carson M. J. Khoury J. E. Landreth G. E. Brosseron F. Feinstein D. L.. (2015). Neuroinflammation in Alzheimer’s disease. Lancet Neurol. 14, 388–405. 10.1016/S1474-4422(15)70016-5 25792098 PMC5909703

[B59] Heneka M. T. Kummer M. P. Latz E. (2014). Innate immune activation in neurodegenerative disease. Nat. Rev. Immunol. 14, 463–477. 10.1038/nri3705 24962261

[B60] Hennessy E. Gormley S. Lopez-Rodriguez A. B. Murray C. Murray C. Cunningham C. (2017). Systemic TNF-α produces acute cognitive dysfunction and exaggerated sickness behavior when superimposed upon progressive neurodegeneration. Brain Behav. Immun. 59, 233–244. 10.1016/j.bbi.2016.09.011 27633985 PMC5176008

[B61] Henry J. Smeyne R. J. Jang H. Miller B. Okun M. S. (2010). Parkinsonism and neurological manifestations of influenza throughout the 20th and 21st centuries. Parkinsonism Relat. Disord. 16, 566–571. 10.1016/j.parkreldis.2010.06.012 20650672 PMC4684089

[B62] Herber D. L. Roth L. M. Wilson D. Wilson N. Mason J. E. Morgan D.. (2004). Time-dependent reduction in Aβ levels after intracranial LPS administration in APP transgenic mice. Exp. Neurol. 190, 245–253. 10.1016/j.expneurol.2004.07.007 15473997

[B63] Herz J. Filiano A. J. Smith A. Yogev N. Kipnis J. (2017). Myeloid cells in the central nervous system. Immunity 46, 943–956. 10.1016/j.immuni.2017.06.007 28636961 PMC5657250

[B64] Hickman S. E. Allison E. K. El Khoury J. (2008). Microglial dysfunction and defective β-amyloid clearance pathways in aging Alzheimer’s disease mice. J. Neurosci. 28, 8354–8360. 10.1523/JNEUROSCI.0616-08.2008 18701698 PMC2597474

[B65] Hickman S. Izzy S. Sen P. Morsett L. El Khoury J. (2018). Microglia in neurodegeneration. Nat. Neurosci. 21, 1359–1369. 10.1038/s41593-018-0242-x 30258234 PMC6817969

[B66] Hines D. J. Hines R. M. Mulligan S. J. Macvicar B. A. (2009). Microglia processes block the spread of damage in the brain and require functional chloride channels. Glia 57, 1610–1618. 10.1002/glia.20874 19382211

[B67] Hoffmann A. Pfeil J. Alfonso J. Kurz F. T. Sahm F. Heiland S.. (2016). Experimental cerebral malaria spreads along the rostral migratory stream. PLoS Pathog. 12:e1005470. 10.1371/journal.ppat.1005470 26964100 PMC4786214

[B68] Hong S. Beja-Glasser V. F. Nfonoyim B. M. Frouin A. Li S. Ramakrishnan S.. (2016). Complement and microglia mediate early synapse loss in Alzheimer mouse models. Science 352, 712–716. 10.1126/science.aad8373 27033548 PMC5094372

[B69] Hosseini S. Wilk E. Michaelsen-Preusse K. Gerhauser I. Baumgärtner W. Geffers R.. (2018). Long-term neuroinflammation induced by influenza a virus infection and the impact on hippocampal neuron morphology and function. J. Neurosci. 38, 3060–3080. 10.1523/JNEUROSCI.1740-17.2018 29487124 PMC6596076

[B70] Hu F. Ku M. C. Markovic D. Dzaye O. D. Lehnardt S. Synowitz M.. (2014). Glioma-associated microglial MMP9 expression is upregulated by TLR2 signaling and sensitive to minocycline. Int. J. Cancer 135, 2569–2578. 10.1002/ijc.28908 24752463 PMC4519695

[B71] Hu H. T. Xiao F. Yan Y. Q. Wen S. Q. Zhang L. (2012). The prognostic value of serum tau in patients with intracerebral hemorrhage. Clin. Biochem. 45, 1320–1324. 10.1016/j.clinbiochem.2012.06.003 22705449

[B72] Ishida N. Ishihara Y. Ishida K. Tada H. Funaki-Kato Y. Hagiwara M.. (2017). Periodontitis induced by bacterial infection exacerbates features of Alzheimer’s disease in transgenic mice. NPJ Aging Mech. Dis. 3:15. 10.1038/s41514-017-0015-x 29134111 PMC5673943

[B73] Itagaki S. McGeer P. L. Akiyama H. (1988). Presence of T-cytotoxic suppressor and leucocyte common antigen positive cells in Alzheimer’s disease brain tissue. Neurosci. Lett. 91, 259–264. 10.1016/0304-3940(88)90690-8 2972943

[B74] Jang H. Boltz D. McClaren J. Pani A. K. Smeyne M. Korff A.. (2012). Inflammatory effects of highly pathogenic H5N1 influenza virus infection in the CNS of mice. J. Neurosci. 32, 1545–1559. 10.1523/JNEUROSCI.5123-11.2012 22302798 PMC3307392

[B75] Janota I. Doshi B. (1979). Cerebral malaria in the United Kingdom. J. Clin. Pathol. 32, 769–772. 10.1136/jcp.32.8.769 389955 PMC1145806

[B76] Jeon H. Lee S. Lee W. H. Suk K. (2010). Analysis of glial secretome: the long pentraxin PTX3 modulates phagocytic activity of microglia. J. Neuroimmunol. 229, 63–72. 10.1016/j.jneuroim.2010.07.001 20674043

[B77] Jin M. Shiwaku H. Tanaka H. Obita T. Ohuchi S. Yoshioka Y.. (2021). Tau activates microglia via the PQBP1-cGAS-STING pathway to promote brain inflammation. Nat. Commun. 12:6565. 10.1038/s41467-021-26851-2 34782623 PMC8592984

[B80] Jurgens H. A. Amancherla K. Johnson R. W. (2012). Influenza infection induces neuroinflammation, alters hippocampal neuron morphology and impairs cognition in adult mice. J. Neurosci. 32, 3958–3968. 10.1523/JNEUROSCI.6389-11.2012 22442063 PMC3353809

[B81] Kahn M. S. Kranjac D. Alonzo C. A. Haase J. H. Cedillos R. O. McLinden K. A.. (2012). Prolonged elevation in hippocampal Aβ and cognitive deficits following repeated endotoxin exposure in the mouse. Behav. Brain Res. 229, 176–184. 10.1016/j.bbr.2012.01.010 22249135

[B82] Kamigaki M. Hide I. Yanase Y. Shiraki H. Harada K. Tanaka Y.. (2016). The Toll-like receptor 4-activated neuroprotective microglia subpopulation survives via granulocyte macrophage colony-stimulating factor and JAK2/STAT5 signaling. Neurochem. Int. 93, 82–94. 10.1016/j.neuint.2016.01.003 26802935

[B83] Kierdorf K. Masuda T. Jordão M. J. C. Prinz M. (2019). Macrophages at CNS interfaces: ontogeny and function in health and disease. Nat. Rev. Neurosci. 20, 547–562. 10.1038/s41583-019-0201-x 31358892

[B84] Kim T. S. Lim H. K. Lee J. Y. Kim D. J. Park S. Lee C.. (2008). Changes in the levels of plasma soluble fractalkine in patients with mild cognitive impairment and Alzheimer’s disease. Neurosci. Lett. 436, 196–200. 10.1016/j.neulet.2008.03.019 18378084

[B85] Kirk R. A. Kesner R. P. Wang L.-M. Wu Q. Towner R. A. Hoffman J. M.. (2019). Lipopolysaccharide exposure in a rat sepsis model results in hippocampal amyloid-β plaque and phosphorylated tau deposition and corresponding behavioral deficits. GeroScience 41, 467–481. 10.1007/s11357-019-00089-9 31473912 PMC6815307

[B86] Kiyofuji K. Kurauchi Y. Hisatsune A. Seki T. Mishima S. Katsuki H. (2015). A natural compound macelignan protects midbrain dopaminergic neurons from inflammatory degeneration via microglial arginase-1 expression. Eur. J. Pharmacol. 760, 129–135. 10.1016/j.ejphar.2015.04.021 25917324

[B87] Kobayashi K. Imagama S. Ohgomori T. Hirano K. Uchimura K. Sakamoto K.. (2013). Minocycline selectively inhibits M1 polarization of microglia. Cell Death Dis. 4:e525. 10.1038/cddis.2013.54 23470532 PMC3613832

[B88] Krabbe G. Halle A. Matyash V. Rinnenthal J. L. Eom G. D. Bernhardt U.. (2013). Functional impairment of microglia coincides with β-amyloid deposition in mice with alzheimer-like pathology. PLoS One 8:e60921. 10.1371/journal.pone.0060921 23577177 PMC3620049

[B89] Krstic D. Madhusudan A. Doehner J. Vogel P. Notter T. Imhof C.. (2012). Systemic immune challenges trigger and drive Alzheimer-like neuropathology in mice. J. Neuroinflammation 9:151. 10.1186/1742-2094-9-151 22747753 PMC3483167

[B90] Kyuwa S. Sugiura Y. (2020). Role of cytotoxic T lymphocytes and interferon-γ in coronavirus infection: lessons from murine coronavirus infections in mice. J. Vet. Med. Sci. 82, 1410–1414. 10.1292/jvms.20-0313 32759577 PMC7653326

[B91] Lacerda-Queiroz N. Brant F. Rodrigues D. H. Vago J. P. Rachid M. A. Sousa L. P.. (2015). Phosphatidylinositol 3-kinase γ is required for the development of experimental cerebral malaria. PLoS One 10:e0119633. 10.1371/journal.pone.0119633 25775137 PMC4361544

[B92] Lajqi T. Lang G. P. Haas F. Williams D. L. Hudalla H. Bauer M.. (2019). Memory-like inflammatory responses of microglia to rising doses of LPS: key role of PI3Kγ. Front. Immunol. 10:2492. 10.3389/fimmu.2019.02492 31781091 PMC6856213

[B93] Lee J. W. Lee Y. K. Yuk D. Y. Choi D. Y. Ban S. B. Oh K. W.. (2008). Neuro-inflammation induced by lipopolysaccharide causes cognitive impairment through enhancement of β-amyloid generation. J. Neuroinflammation 5:37. 10.1186/1742-2094-5-37 18759972 PMC2556656

[B94] Lee S. Varvel N. H. Konerth M. E. Xu G. Cardona A. E. Ransohoff R. M.. (2010). CX3CR1 deficiency alters microglial activation and reduces β-amyloid deposition in two Alzheimer’s disease mouse models. Am. J. Pathol. 177, 2549–2562. 10.2353/ajpath.2010.100265 20864679 PMC2966811

[B95] Leem Y. H. Park J. S. Park J. E. Kim D. Y. Kim H. S. (2021). Papaverine exerts neuroprotective effect by inhibiting NLRP3 inflammasome activation in an mptp-induced microglial priming mouse model challenged with LPS. Biomol. Ther. 29, 295–302. 10.4062/biomolther.2021.039 33911050 PMC8094076

[B96] Leibovitch E. C. Jacobson S. (2014). Evidence linking HHV-6 with multiple sclerosis: an update. Curr. Opin. Virol. 9, 127–133. 10.1016/j.coviro.2014.09.016 25462444 PMC4269240

[B97] Lenz K. M. McCarthy M. M. (2015). A starring role for microglia in brain sex differences. Neuroscientist 21:306. 10.1177/1073858414536468 24871624 PMC5742269

[B98] Li W. Y. Chang Y. C. Lee L. J. H. Lee L. J. (2014). Prenatal infection affects the neuronal architecture and cognitive function in adult mice. Dev. Neurosci. 36, 359–370. 10.1159/000362383 24942872

[B99] Lyons A. Lynch A. M. Downer E. J. Hanley R. O’Sullivan J. B. Smith A.. (2009). Fractalkine-induced activation of the phosphatidylinositol-3 kinase pathway attentuates microglial activation *in vivo* and *in vitro*. J. Neurochem. 110, 1547–1556. 10.1111/j.1471-4159.2009.06253.x 19627440

[B100] Mahmoudvand H. Sheibani V. Shojaee S. Mirbadie S. R. Keshavarz H. Esmaeelpour K.. (2016). Toxoplasma gondii infection potentiates cognitive impairments of Alzheimer’s disease in the BALB/c mice. J. Parasitol. 102, 629–635. 10.1645/16-28 27513205

[B101] Maniega S. M. Valdés Hernández M. C. Clayden J. D. Royle N. A. Murray C. Morris Z.. (2015). White matter hyperintensities and normal-appearing white matter integrity in the aging brain. Neurobiol. Aging 36, 909–918. 10.1016/j.neurobiolaging.2014.07.048 25457555 PMC4321830

[B102] Markovic D. S. Vinnakota K. van Rooijen N. Kiwit J. Synowitz M. Glass R.. (2011). Minocycline reduces glioma expansion and invasion by attenuating microglial MT1-MMP expression. Brain. Behav. Immun. 25, 624–628. 10.1016/j.bbi.2011.01.015 21324352

[B103] Marschallinger J. Iram T. Zardeneta M. Lee S. E. Lehallier B. Haney M. S.. (2020). Lipid-droplet-accumulating microglia represent a dysfunctional and proinflammatory state in the aging brain. Nat. Neurosci. 23, 194–208. 10.1038/s41593-019-0566-1 31959936 PMC7595134

[B104] Marttinen M. Takalo M. Natunen T. Wittrahm R. Gabbouj S. Kemppainen S.. (2018). Molecular mechanisms of synaptotoxicity and neuroinflammation in Alzheimer’s disease. Front. Neurosci. 12:963. 10.3389/fnins.2018.00963 30618585 PMC6301995

[B105] Matschke J. Lütgehetmann M. Hagel C. Sperhake J. P. Schröder A. S. Edler C.. (2020). Neuropathology of patients with COVID-19 in Germany: a post-mortem case series. Lancet Neurol. 19, 919–929. 10.1016/S1474-4422(20)30308-2 33031735 PMC7535629

[B106] Mattock C. Marmot M. Stern G. (1988). Could Parkinson’s disease follow intra-uterine influenza?: a speculative hypothesis. J. Neurol. Neurosurg. Psychiatry 51, 753–756. 10.1136/jnnp.51.6.753 3404182 PMC1033143

[B107] Mattsson N. Zetterberg H. Nielsen N. Blennow K. Dankiewicz J. Friberg H.. (2017). Serum tau and neurological outcome in cardiac arrest. Ann. Neurol. 82, 665–675. 10.1002/ana.25067 28981963 PMC5725735

[B108] Mawuenyega K. G. Sigurdson W. Ovod V. Munsell L. Kasten T. Morris J. C.. (2010). Decreased clearance of CNS β-amyloid in Alzheimer’s disease. Science 330:1774. 10.1126/science.1197623 21148344 PMC3073454

[B109] Mbagwu S. I. Lannes N. Walch M. Filgueira L. Mantel P. Y. (2020). Human microglia respond to Malaria-induced extracellular vesicles. Pathogens 9:21. 10.3390/pathogens9010021 31878288 PMC7168629

[B110] McGeer P. L. Itagaki S. Boyes B. E. McGeer E. G. (1988). Reactive microglia are positive for HLA-DR in the: substantia nigra of Parkinson’s and Alzheimer’s disease brains. Neurology 38, 1285–1291. 10.1212/wnl.38.8.1285 3399080

[B111] McGeer P. L. Itagaki S. Tago H. McGeer E. G. (1987). Reactive microglia in patients with senile dementia of the Alzheimer type are positive for the histocompatibility glycoprotein HLA-DR. Neurosci. Lett. 79, 195–200. 10.1016/0304-3940(87)90696-3 3670729

[B112] Meyer-Luehmann M. Spires-Jones T. L. Prada C. Garcia-Alloza M. De Calignon A. Rozkalne A.. (2008). Rapid appearance and local toxicity of amyloid-β plaques in a mouse model of Alzheimer’s disease. Nature 451, 720–724. 10.1038/nature06616 18256671 PMC3264491

[B113] Miller D. B. O’Callaghan J. P. (2008). Do early-life insults contribute to the late-life development of Parkinson and Alzheimer diseases? Metabolism 57, S44–S49. 10.1016/j.metabol.2008.07.011 18803966

[B114] Mizuno T. (2015). Neuron-microglia interactions in neuroinflammation. Clin. Exp. Neuroimmunol. 6, 225–231. 10.1111/cen3.12228 PMC4629520 26543505

[B115] Moreno B. Jukes J. P. Vergara-Irigaray N. Errea O. Villoslada P. Perry V. H.. (2011). Systemic inflammation induces axon injury during brain inflammation. Ann. Neurol. 70, 932–942. 10.1002/ana.22550 22190366

[B116] Murray C. L. Skelly D. T. Cunningham C. (2011). Exacerbation of CNS inflammation and neurodegeneration by systemic LPS treatment is independent of circulating IL-1β and IL-6. J. Neuroinflammation 8:50. 10.1186/1742-2094-8-50 21586125 PMC3119173

[B117] Muzio L. Viotti A. Martino G. (2021). Microglia in neuroinflammation and neurodegeneration: from understanding to therapy. Front. Neurosci. 15:1205. 10.3389/fnins.2021.742065 34630027 PMC8497816

[B118] Nakada T. A. Tomita K. Oshima T. Kawaguchi R. Oda S. (2020). Serum levels of tau protein increase according to the severity of the injury in DAI rat model. F1000Research 9:29. 10.12688/f1000research.21132.1 33299544 PMC7707115

[B119] Nalivaeva N. N. Beckett C. Belyaev N. D. Turner A. J. (2012). Are amyloid-degrading enzymes viable therapeutic targets in Alzheimer’s disease? J. Neurochem. 120, 167–185. 10.1111/j.1471-4159.2011.07510.x 22122230

[B120] Neher J. J. Neniskyte U. Zhao J.-W. Bal-Price A. Tolkovsky A. M. Brown G. C.. (2011). Inhibition of microglial phagocytosis is sufficient to prevent inflammatory neuronal death. J. Immunol. 186, 4973–4983. 10.4049/jimmunol.1003600 21402900

[B121] Nimmerjahn A. Kirchhoff F. Helmchen F. (2005). Neuroscience: resting microglial cells are highly dynamic surveillants of brain parenchyma *in vivo*. Science 308, 1314–1318. 10.1126/science.1110647 15831717

[B122] Ogata A. Tashiro K. Nukuzuma S. Nagashima K. Hall W. W. (1997). A rat model of Parkinson’s disease induced by Japanese encephalitis virus. J. Neurovirol. 3, 141–147. 10.3109/13550289709015803 9111176

[B123] Osborne B. F. Caulfield J. I. Solomotis S. A. Schwarz J. M. (2017). Neonatal infection produces significant changes in immune function with no associated learning deficits in juvenile rats. Dev. Neurobiol. 77, 1221–1236. 10.1002/dneu.22512 28719141 PMC5777507

[B124] Oxford J. S. (2000). Influenza a pandemics of the 20th century with special reference to 1918: virology, pathology and epidemiology. Rev. Med. Virol. 10, 119–133. 10.1002/(sici)1099-1654(200003/04)10:2<119::aid-rmv272>3.0.co;2-o 10713598

[B125] Palmio J. Suhonen J. Keränen T. Hulkkonen J. Peltola J. Pirttilä T.. (2009). Cerebrospinal fluid tau as a marker of neuronal damage after epileptic seizure. Seizure 18, 474–477. 10.1016/j.seizure.2009.04.006 19428269

[B126] Paolicelli R. Sierra A. Stevens B. Tremblay M.-E. Aguzzi A. Ajami B.. (2022). Defining microglial states and nomenclature: a roadmap to 2030. SSRN Electron. J. [Preprint]. 10.2139/ssrn.4065080

[B127] Perea J. R. Bolós M. Avila J. (2020). Microglia in Alzheimer’s disease in the context of tau pathology. Biomolecules 10:1439. 10.3390/biom10101439 33066368 PMC7602223

[B129] Perea J. R. Llorens-Martín M. Ávila J. Bolós M. (2018a). The role of microglia in the spread of Tau: relevance for tauopathies. Front. Cell. Neurosci. 12:172. 10.3389/fncel.2018.00172 30042659 PMC6048186

[B128] Perea J. R. Lleó A. Alcolea D. Fortea J. Ávila J. Bolós M. (2018b). Decreased CX3CL1 levels in the cerebrospinal fluid of patients with Alzheimer’s disease. Front. Neurosci. 12:609. 10.3389/fnins.2018.00609 30245615 PMC6137321

[B130] Perry V. H. Holmes C. (2014). Microglial priming in neurodegenerative disease. Nat. Rev. Neurol. 10, 217–224. 10.1038/nrneurol.2014.38 24638131

[B131] Perry V. H. Teeling J. (2013). Microglia and macrophages of the central nervous system: the contribution of microglia priming and systemic inflammation to chronic neurodegeneration. Semin. Immunopathol. 35, 601–612. 10.1007/s00281-013-0382-8 23732506 PMC3742955

[B132] Perry V. H. Cunningham C. Holmes C. (2007). Systemic infections and inflammation affect chronic neurodegeneration. Nat. Rev. Immunol. 7, 161–167. 10.1038/nri2015 17220915

[B133] Plescher M. Seifert G. Hansen J. N. Bedner P. Steinhäuser C. Halle A. (2018). Plaque-dependent morphological and electrophysiological heterogeneity of microglia in an Alzheimer’s disease mouse model. Glia 66, 1464–1480. 10.1002/glia.23318 29493017

[B134] Püntener U. Booth S. G. Perry V. H. Teeling J. L. (2012). Long-term impact of systemic bacterial infection on the cerebral vasculature and microglia. J. Neuroinflammation 9:146. 10.1186/1742-2094-9-146 22738332 PMC3439352

[B135] Quan N. Whiteside M. Herkenham M. (1998). Time course and localization patterns of interleukin-1β messenger RNA expression in brain and pituitary after peripheral administration of lipopolysaccharide. Neuroscience 83, 281–293. 10.1016/s0306-4522(97)00350-3 9466417

[B136] Rajbhandari L. Tegenge M. A. Shrestha S. Ganesh Kumar N. Malik A. Mithal A.. (2014). Toll-like receptor 4 deficiency impairs microglial phagocytosis of degenerating axons. Glia 62, 1982–1991. 10.1002/glia.22719 25042766

[B137] Randall J. Mörtberg E. Provuncher G. K. Fournier D. R. Duffy D. C. Rubertsson S.. (2013). Tau proteins in serum predict neurological outcome after hypoxic brain injury from cardiac arrest: results of a pilot study. Resuscitation 84, 351–356. 10.1016/j.resuscitation.2012.07.027 22885094

[B138] Ratnayake U. Quinn T. Walker D. W. Dickinson H. (2013). Cytokines and the neurodevelopmental basis of mental illness. Front. Neurosci. 7:180. 10.3389/fnins.2013.00180 24146637 PMC3797953

[B139] Ravenholt R. T. Foege W. H. (1982). 1918 Influenza, encephalitis lethargica, parkinsonism. Lancet 320, 860–864. 10.1016/s0140-6736(82)90820-0 6126720

[B140] Readhead B. Haure-Mirande J. V. Funk C. C. Richards M. A. Shannon P. Haroutunian V.. (2018). Multiscale analysis of independent Alzheimer’s cohorts finds disruption of molecular, genetic and clinical networks by human herpesvirus. Neuron 99, e764–e782. 10.1016/j.neuron.2018.05.023 29937276 PMC6551233

[B141] Reeve A. Simcox E. Turnbull D. (2014). Ageing and Parkinson’s disease: why is advancing age the biggest risk factor? Ageing Res. Rev. 14, 19–30. 10.1016/j.arr.2014.01.004 24503004 PMC3989046

[B142] Ries M. Sastre M. (2016). Mechanisms of Aβ clearance and degradation by glial cells. Front. Aging Neurosci. 8:160. 10.3389/fnagi.2016.00160 27458370 PMC4932097

[B143] Romero C. R. Herzig D. S. Etogo A. Nunez J. Mahmoudizad R. Fang G.. (2010). The role of interferon-γ in the pathogenesis of acute intra-abdominal sepsis. J. Leukoc. Biol. 88, 725–735. 10.1189/jlb.0509307 20628064 PMC2974432

[B144] Sadasivan S. Sharp B. Schultz-Cherry S. Smeyne R. J. (2017). Synergistic effects of influenza and 1-methyl-4-phenyl-1,2,3,6-tetrahydropyridine (MPTP) can be eliminated by the use of influenza therapeutics: experimental evidence for the multi-hit hypothesis. NPJ Park. Dis. 3:18. 10.1038/s41531-017-0019-z 28649618 PMC5460228

[B145] Sadasivan S. Zanin M. O’Brien K. Schultz-Cherry S. Smeyne R. J. (2015). Induction of microglia activation after infection with the non-neurotropic A/CA/04/2009 H1N1 influenza virus. PLoS One 10:e0124047. 10.1371/journal.pone.0124047 25861024 PMC4393251

[B146] Sasaki A. Kawarabayashi T. Murakami T. Matsubara E. Ikeda M. Hagiwara H.. (2008). Microglial activation in brain lesions with tau deposits: comparison of human tauopathies and tau transgenic mice TgTauP301L. Brain Res. 1214, 159–168. 10.1016/j.brainres.2008.02.084 18457819

[B147] Savage J. C. St-Pierre M. K. Hui C. W. Tremblay M. E. (2019). Microglial ultrastructure in the hippocampus of a lipopolysaccharide-induced sickness mouse model. Front. Neurosci. 13:1340. 10.3389/fnins.2019.01340 31920505 PMC6932978

[B148] Scheffel J. Regen T. Van Rossum D. Seifert S. Ribes S. Nau R.. (2012). Toll-like receptor activation reveals developmental reorganization and unmasks responder subsets of microglia. Glia 60, 1930–1943. 10.1002/glia.22409 22911652

[B149] Schiess N. Villabona-Rueda A. Cottier K. E. Huether K. Chipeta J. Stins M. F.. (2020). Pathophysiology and neurologic sequelae of cerebral malaria. Malar. J. 19:266. 10.1186/s12936-020-03336-z 32703204 PMC7376930

[B150] Schluesener H. J. Kremsner P. G. Meyermann R. (1998). Widespread expression of MRP8 and MRP14 in human cerebral malaria by microglial cells. Acta Neuropathol. 96, 575–580. 10.1007/s004010050938 9845287

[B151] Schroder K. Sweet M. J. Hume D. A. (2006). Signal integration between IFNγ and TLR signalling pathways in macrophages. Immunobiology 211, 511–524. 10.1016/j.imbio.2006.05.007 16920490

[B152] Shabab T. Khanabdali R. Moghadamtousi S. Z. Kadir H. A. Mohan G. (2017). Neuroinflammation pathways: a general review. Int. J. Neurosci. 127, 624–633. 10.1080/00207454.2016.1212854 27412492

[B153] Shaerzadeh F. Phan L. Miller D. Dacquel M. Hachmeister W. Hansen C.. (2020). Microglia senescence occurs in both substantia nigra and ventral tegmental area. Glia 68, 2228–2245. 10.1002/glia.23834 32275335 PMC8356201

[B154] Sierra A. Gottfried-Blackmore A. C. Mcewen B. S. Bulloch K. (2007). Microglia derived from aging mice exhibit an altered inflammatory profile. Glia 55, 412–424. 10.1002/glia.20468 17203473

[B155] Simpson D. S. A. Oliver P. L. (2020). ROS generation in microglia: understanding oxidative stress and inflammation in neurodegenerative disease. Antioxidants (Basel) 9:743. 10.3390/antiox9080743 32823544 PMC7463655

[B156] Sly L. M. Krzesicki R. F. Brashler J. R. Buhl A. E. McKinley D. D. Carter D. B.. (2001). Endogenous brain cytokine mRNA and inflammatory responses to lipopolysaccharide are elevated in the Tg2576 transgenic mouse model of Alzheimer’s disease. Brain Res. Bull. 56, 581–588. 10.1016/s0361-9230(01)00730-4 11786245

[B157] Souza T. L. D. Grauncke A. C. B. Ribeiro L. R. Mello F. K. Oliveira S. M. Brant F.. (2018). Cerebral malaria causes enduring behavioral and molecular changes in mice brain without causing gross histopathological damage. Neuroscience 369, 66–75. 10.1016/j.neuroscience.2017.10.043 29113928

[B158] Španić E. Langer Horvat L. Hof P. R. Šimić G. (2019). Role of microglial cells in Alzheimer’s disease tau propagation. Front. Aging Neurosci. 11:271. 10.3389/fnagi.2019.00271 31636558 PMC6787141

[B159] Spencer N. G. Schilling T. Miralles F. Eder C. (2016). Mechanisms underlying interferon-γ-induced priming of microglial reactive oxygen species production. PLoS One 11:e0162497. 10.1371/journal.pone.0162497 27598576 PMC5012572

[B160] Stratoulias V. Venero J. L. Tremblay M. Joseph B. (2019). Microglial subtypes: diversity within the microglial community. EMBO J. 38:e101997. 10.15252/embj.2019101997 31373067 PMC6717890

[B161] Streit W. J. (2002). Microglia as neuroprotective, immunocompetent cells of the CNS. Glia 40, 133–139. 10.1002/glia.10154 12379901

[B162] Sturge C. R. Yarovinsky F. (2014). Complex immune cell interplay in the gamma interferon response during *Toxoplasma gondii* infection. Infect. Immun. 82, 3090–3097. 10.1128/IAI.01722-14 24866795 PMC4136216

[B163] Subhramanyam C. S. Wang C. Hu Q. Dheen S. T. (2019). Microglia-mediated neuroinflammation in neurodegenerative diseases. Semin. Cell Dev. Biol. 94, 112–120. 10.1016/j.semcdb.2019.05.004 31077796

[B164] Tahira A. C. Verjovski-Almeida S. Ferreira S. T. (2021). Dementia is an age-independent risk factor for severity and death in COVID-19 inpatients. Alzheimers. Dement. 17, 1818–1831. 10.1002/alz.12352 33881211 PMC8250282

[B165] Talavera-López C. Capuccini B. Mitter R. Lin J. W. Langhorne J. (2018). Transcriptomes of microglia in experimental cerebral malaria in mice in the presence and absence of Type I interferon signaling. BMC Res. Notes 11:913. 10.1186/s13104-018-4020-3 30572937 PMC6302474

[B166] Tang Y. Liu H. L. Min L. X. Yuan H. S. Guo L. Han P. B.. (2019). Serum and cerebrospinal fluid tau protein level as biomarkers for evaluating acute spinal cord injury severity and motor function outcome. Neural Regen. Res. 14, 896–902. 10.4103/1673-5374.249238 30688276 PMC6375043

[B167] Tay T. L. Savage J. C. Hui C. W. Bisht K. Tremblay M. È. (2017). Microglia across the lifespan: from origin to function in brain development, plasticity and cognition. J. Physiol. 595, 1929–1945. 10.1113/JP272134 27104646 PMC5350449

[B168] Tejera D. Mercan D. Sanchez-Caro J. M. Hanan M. Greenberg D. Soreq H.. (2019). Systemic inflammation impairs microglial Aβ clearance through NLRP 3 inflammasome. EMBO J. 38:e101064. 10.15252/embj.2018101064 31359456 PMC6717897

[B169] Therajaran P. Hamilton J. A. O’Brien T. J. Jones N. C. Ali I. (2020). Microglial polarization in posttraumatic epilepsy: potential mechanism and treatment opportunity. Epilepsia 61, 203–215. 10.1111/epi.16424 31943156

[B170] Vainchtein I. D. Molofsky A. V. (2020). Astrocytes and microglia: in sickness and in health. Trends Neurosci. 43, 144–154. 10.1016/j.tins.2020.01.003 32044129 PMC7472912

[B171] Velagapudi R. Kosoko A. M. Olajide O. A. (2019). Induction of neuroinflammation and neurotoxicity by synthetic hemozoin. Cell. Mol. Neurobiol. 39, 1187–1200. 10.1007/s10571-019-00713-4 31332667 PMC6764936

[B79] Vilensky J. A. Gilman S. McCall S. (2010). A historical analysis of the relationship between encephalitis lethargica and postencephalitic parkinsonism: a complex rather than a direct relationship. Mov. Disord. 25, 1116–1123. 10.1002/mds.22908 20629120

[B172] Villa A. Della Torre S. Maggi A. (2019). Sexual differentiation of microglia. Front. Neuroendocrinol. 52, 156–164. 10.1016/j.yfrne.2018.11.003 30481522

[B173] Villoslada P. Baeza-Yates R. Masdeu J. C. (2020). Reclassifying neurodegenerative diseases. Nat. Biomed. Eng. 4, 759–760. 10.1038/s41551-020-0600-3 32747833

[B174] Vitkovic L. Konsman J. P. Bockaert J. Dantzer R. Homburger V. Jacque C.. (2000). Cytokine signals propagate through the brain. Mol. Psychiatry 5, 604–615. 10.1038/sj.mp.4000813 11126391

[B176] Wang J.-Z. Xia Y.-Y. Grundke-Iqbal I. Iqbal K. (2013). Abnormal hyperphosphorylation of tau: sites, regulation and molecular mechanism of neurofibrillary degeneration. J. Alzheimer’s Dis. 33, S123–S139. 10.3233/JAD-2012-129031 22710920

[B175] Wang F. Zhang Z. Z. Cao L. Yang Q. G. Lu Q. F. Chen G. H.. (2020). Lipopolysaccharide exposure during late embryogenesis triggers and drives Alzheimer-like behavioral and neuropathological changes in CD-1 mice. Brain Behav. 10:e01546. 10.1002/brb3.1546 31997558 PMC7066339

[B177] Wendeln A. C. Degenhardt K. Kaurani L. Gertig M. Ulas T. Jain G.. (2018). Innate immune memory in the brain shapes neurological disease hallmarks. Nature 556, 332–338. 10.1038/s41586-018-0023-4 29643512 PMC6038912

[B178] Widmann C. N. Heneka M. T. (2014). Long-term cerebral consequences of sepsis. Lancet Neurol. 13, 630–636. 10.1016/S1474-4422(14)70017-1 24849863

[B179] Wynne A. M. Henry C. J. Godbout J. P. (2009). Immune and behavioral consequences of microglial reactivity in the aged brain. Integr. Comp. Biol. 49, 254–266. 10.1093/icb/icp009 21665818 PMC4447842

[B180] Wynne A. M. Henry C. J. Huang Y. Cleland A. Godbout J. P. (2010). Protracted downregulation of CX3CR1 on microglia of aged mice after lipopolysaccharide challenge. Brain Behav. Immun. 24, 1190–1201. 10.1016/j.bbi.2010.05.011 20570721 PMC2939290

[B181] Yeini E. Ofek P. Pozzi S. Albeck N. Ben-Shushan D. Tiram G.. (2021). P-selectin axis plays a key role in microglia immunophenotype and glioblastoma progression. Nat. Commun. 12:1912. 10.1038/s41467-021-22186-0 33771989 PMC7997963

[B182] Young G. B. (2013). Encephalopathy of infection and systemic inflammation. J. Clin. Neurophysiol. 30, 454–461. 10.1097/WNP.0b013e3182a73d83 24084178

[B183] Zhao T. Xia Y. Wang D. Pang L. (2019). Association between elevated serum tau protein level and sepsis-associated encephalopathy in patients with severe sepsis. Can. J. Infect. Dis. Med. Microbiol. 2019:1876174. 10.1155/2019/1876174 31396296 PMC6664571

[B184] Zilka N. Stozicka Z. Kovac A. Pilipcinec E. Bugos O. Novak M.. (2009). Human misfolded truncated tau protein promotes activation of microglia and leukocyte infiltration in the transgenic rat model of tauopathy. J. Neuroimmunol. 209, 16–25. 10.1016/j.jneuroim.2009.01.013 19232747

